# From Late Miocene to Holocene: Processes of Differentiation within the *Telestes* Genus (Actinopterygii: Cyprinidae)

**DOI:** 10.1371/journal.pone.0034423

**Published:** 2012-03-29

**Authors:** Vincent Dubut, Antoine Fouquet, Adrien Voisin, Caroline Costedoat, Rémi Chappaz, André Gilles

**Affiliations:** 1 Aix-Marseille Université, CNRS, IRD, UMR 7263 – IMBE, Equipe Evolution Génome Environnement, Centre Saint-Charles, Case 36, Marseille, France; 2 CNRS, USR 3456 ‘CNRS-Guyane’, Immeuble Le Relais, Cayenne, Guyane Française; University of York, United Kingdom

## Abstract

Investigating processes and timing of differentiation of organisms is critical in the understanding of the evolutionary mechanisms involved in microevolution, speciation, and macroevolution that generated the extant biodiversity. From this perspective, the *Telestes* genus is of special interest: the *Telestes* species have a wide distribution range across Europe (from the Danubian district to Mediterranean districts) and have not been prone to translocation. Molecular data (mtDNA: 1,232 bp including the entire Cyt *b* gene; nuclear genome: 11 microsatellites) were gathered from 34 populations of the *Telestes* genus, almost encompassing the entire geographic range. Using several phylogenetic and molecular dating methods interpreted in conjunction with paleoclimatic and geomorphologic evidence, we investigated the processes and timing of differentiation of the *Telestes* lineages. The observed genetic structure and diversity were largely congruent between mtDNA and microsatellites. The Messinian Salinity Crisis (Late Miocene) seems to have played a major role in the speciation processes of the genus. Focusing on *T. souffia*, a species occurring in the Danube and Rhone drainages, we were able to point out several specific events from the Pleistocene to the Holocene that have likely driven the differentiation and the historical demography of this taxon. This study provides support for an evolutionary history of dispersal and vicariance with unprecedented resolution for any freshwater fish in this region.

## Introduction

Three analytical levels can be defined when dealing with the processes of evolutionary differentiation [1]: microevolution (within and among populations), speciation, and supra-specific differentiation (macroevolution). One of the central questions of the study of genetic differentiation is to understand the putative barriers (preventing gene flow between populations) involved at these three levels [2]. The nature of the geomorphologic events that have fostered speciation and shape population structure of freshwater fishes is still strongly debated [3], [4]. A number of causes have been suggested, including (i) watersheds crossing by upstream river capture, (ii) downstream river connections (due to modification of the sea level), (iii) transient tributary connections (e.g. due to floods), (iv) periglacial lakes as vectors for colonisation processes, and (v) subsidence lakes.

For European freshwater fishes, these geomorphologic events are usually related to (i) the Messinian Salinity Crisis (MSC [5]), which started 5.96 Myr by the end of the Late Miocene [6] and was followed by the Lago Mare stage of the Mediterranean [5] between 5.50 and 5.33 Myr [6], or (ii) the Quaternary (from 1.8 Myr to present) glaciation cycles. The MSC and its final Lago Mare stage are considered as major events in the speciation and dispersal of freshwater fish taxa in the peri-Mediterranean area [7]–[9]. While the climatic oscillations of the Pleistocene are often invoked as major events in the formation of the geographic and genetic structure of current species via past range fragmentation, population contraction and re-colonisation (e.g. [10]–[12]).

The processes of colonisation of freshwater European ichthyofauna have received particular attention during the last decade, especially in Cyprinids. Many studies based on mitochondrial DNA (mtDNA) explored the major diversifications of this family in Europe and proposed an array of evolutionary scenarios for species or groups of species (reviewed in [13]). Other studies, which focused on finer scale analyses and were generally based on microsatellites, provided elements of the more recent evolutionary history of European ichthyofauna (e.g. [14]). The relative impact and importance of the MSC and the Pleistocene glaciations in the differentiation processes of European freshwater fishes is still debated (e.g. [15], [16]). More importantly, studies investigating the influence of Pleistocene glaciations are generally limited to challenging the impact of the Last Glacial Maximum (LGM) ∼20 Kyr ago. The detailed dynamics of demographic contraction, fragmentation and re-colonisation processes of European freshwater species during the Pleistocene remains poorly understood (but see [17], [18]). As a matter of fact, the Pleistocene has been shown to have been a critical period for the establishment of the present day genetic diversity of terrestrial fauna (e.g. [19], [20]). However, the Pleistocene (1.8 Myr) consisted of a series of well characterized glacials and interglacials [21] that have not systematically erased the prints and distribution patterns inherited from previous cycles but may, alternatively, have added new layers of complexity. Therefore, deciphering the relative importance of the successive historical events that may have shaped current species distribution and their genetic structure is challenging as it requires extensive sampling effort and enough data informativeness.

From this perspective, the *Telestes* genus is of special interest. A taxonomic revision and a thorough look at the differentiation pattern based on partial cytochrome *b* sequence, allozymes and morphology were recently undertaken for the *Telestes* genus [22], providing a solid foundation to investigate processes of differentiation in this genus. This study also suggested that the *Telestes* genus speciated mostly via allopatric isolation and that introgressive hybridization only had a marginal role [22]. Furthermore, the *Telestes* genus constitutes an attractive freshwater fish model considering its peculiar ecological niche. For instance, *T. souffia* generally occurs in ∼5 m wide rivers from ∼10 km downstream of the source [23], [24]. The mean discharge has no significant influence on its distribution and the relevant summer temperature varies between 11°C and 26.5°C [23], [24]. The species is rare or absent in large rivers [23] and dispersal is believed to be limited [25]. *Telestes souffia* is therefore considered as adapted to relatively small running rivers and cold-water. It is also noteworthy that species of the *Telestes* genus are rather small bodied (max. 160 mm). These species are thus not valuable for fishing and are therefore less prone to be translocated, an activity that is well known in economically valuable freshwater fish species [26]–[28] to alter original population structure [29].

Using 970 specimens, almost encompassing the entire geographic range of *Telestes*, we gathered molecular data from a 1232 bp long mitochondrial sequence (including the complete cytochrome *b* gene and 92 bp of flanking tRNA genes) and 11 nuclear microsatellite loci to investigate the major mechanisms and events responsible for the genetic structure and diversity pattern in *Telestes*. We explored whether (i) the MSC/Lago Mare event, (ii) the Pleistocene glaciation cycles, and/or (iii) the post-LGM/Holocene period could account for the current species distribution pattern and their genetic structure. Furthermore, we stressed the importance of watersheds crossing by upstream river capture periglacial lakes and downstream river connections in the *Telestes* genus evolution.

## Materials and Methods

### Ethics Statement

This study was conducted according to relevant national and international guidelines regarding the care and welfare of fishes. Field studies did not involve fish that were endangered or protected (The IUCN Red List of Threatened Species v. 2011.1; www.iucnredlist.org). The fishes collected for this study were killed rapidly (using clove oil as anesthetic) or were returned to the wild. The DDTs (Directions Départementales des Territoires) from the Alpes-de-Haute-Provence, Hautes-Alpes, Vaucluse, Ardèche, Haute-Savoie, Doubs, Ain and Saone districts issued the permits for France, where fishing was done in collaboration with Officers of the ONEMA (Office National de l'Eau et des Milieux Aquatiques, France). Permits for the localities in Romania, Italy, Greece and Montenegro were supplied respectively by the Institute of Biology of Bucarest (representative Petru Bănărescu), the Universita di Napoli ‘Federico II’ (representative Pier Giorgio Bianco), the Aristotle University, Thessaloniki (representative Panos Stavros Economidis) and the University of Montenegro, Podgorica (representative Drago Marić). The permit for the Slovenian sampling was supplied by the Angler Society of Idrija, the Angler Society of Sevnica and the Fisheries Research Institute of Ljubljana as part of the research project 9E0073 funded entirely by the Tour du Valat Foundation (representative Alain Crivelli) and the WWF International.

### Sampling and DNA protocols

**Figure 1:**
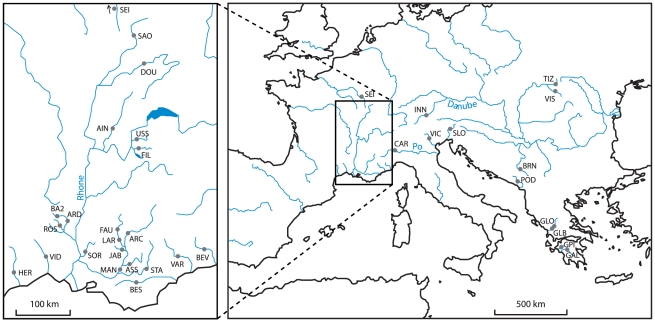
Location of the sampled *Telestes* populations with emphasize on the Rhone and French Mediterranean basins. For samples ID, see Table 1.

A total of 970 individuals from 34 locations were collected for this study (Fig. 1), covering almost the entire geographical range of the *Telestes* genus. The 34 locations encompassed six European biogeographical districts (as defined by [8], [30]): the Western-Greece district (WG), the Albanian district (AB), the Padano-Venetian district (PV), the Tuscano-Latium district (TL), the Danubian district (DB) and the Southern France district (SF). A more detailed sampling was performed in the Rhone drainage (in SF district) in order to conduct a finer scale analysis of the pattern of genetic variation in this area. Further details on samples are reported in Figure 1 and Table 1.

**Table 1:** Sampled *Telestes* taxa, population locations and sample sizes.

Taxa	Sample ID	Sampling year	River	River basin (sub-basin)	District^a^	mtDNA	Microsatellites
*Telestes alfiensis*	GAL	2003	Alfios	Alfios	WG	4	4
	GPI	2003	Pinios	Pinios	WG	1	1
*Telestes pleurobipunctatus*	GLO	2003	Louros Ms	Louros	WG	9	9
	GLB	2003	Louros Ps	Louros	WG	23	22
*Telestes montenigrinus*	POD	2001	Zeta	Moraca	AB	30	31
*Telestes muticellus*	CAR	1997	Po	Po	PV	29	25
	VIC	1997	Bacchiglione	Bacchiglione	PV	27	24
	BEV	1996	Bevera	Roya	TL	29	29
*Teleste souffia agassii* 1	BRN	2001	Lim	Danube	DB	31	28
	SLO	2000	Soca	Soca	PV	38	37
*Teleste souffia agassii* 2	TIZ	1999	Tisza	Danube	DB	21	15
	VIS	1999	Viseu	Danube	DB	24	15
*Telestes souffia agassii* 3	INN	2002	Inn	Danube	DB	28	30
*Telestes souffia souffia*	AIN	1996	Ain	Rhone	SF	29	28
	ARC	1996	Durance	Rhone (Durance)	SF	30	29
	ARD	1996	Ardeche	Rhone (Ardeche)	SF	20	20
	ASS	1996	Asse	Rhone (Durance)	SF	39	28
	BA2	1996	Ardeche	Rhone (Ardeche)	SF	30	29
	BES	1996	Argens	Argens	SF	23	23
	DOU	1996	Doubs	Rhone	SF	33	23
	FAU	1996	Buech	Rhone (Durance)	SF	29	28
	FIL	1996	Filliere	Rhone	SF	31	33
	HER	1996	Herault	Herault	SF	33	33
	JAB	1996	Jabron	Rhone (Durance)	SF	22	21
	LAR	1996	Buech	Rhone (Durance)	SF	43	42
	MAN	1996	Durance	Rhone (Durance)	SF	22	20
	ROS	2008	Beaume	Rhone (Ardeche)	SF	39	39
	SAO	1996	Saone	Rhone	SF	30	24
	SEI	1996	Ource	Seine	DB	26	23
	SOR	1996	Sorgue	Rhone	SF	30	29
	STA	1996	Verdon	Rhone (Durance)	SF	35	28
	USS	1996	Usses	Rhone	SF	34	24
	VAR	1996	Var	Var	SF	67	65
	VID	1996	Vidourle	Vidourle	SF	31	30

a see main text for abbreviations.

Whole DNA was extracted from 25 mg of muscle or 0.25 cm^2^ of caudal fin using the Gentra® Puregen™ Tissue Kit (QIAGEN) following the manufacturer's instructions. A 1275 bp mitochondrial DNA (mtDNA) coding region was amplified by PCR using the *Taq* CORE Kit (MP Biomedicals) with primers L14350C (5′-ACCACCGTTGTAGTTCAACTAC-3′) and H15620U (5′-AGGGGTGGGAGTTAAAATCTC-3′). PCRs were conducted following standard conditions in a total volume of 40 µL with 1 U of *Taq* polymerase and 2 µL of 1∶10 diluted DNA extract (PCR cycling protocol: initial denaturation at 95°C for 5 min; denaturation at 95°C for 30 s, annealing at 60°C for 30 s and extension at 72°C for 1 min 30 s, repeated for 35 cycles; final extension at 72°C for 10 min). The amplicons were sequenced by GATC Biotech (Konstanz, Germany) using internal primers Cb-L500 (5′-CAATGAATCTGAGGCGGTTT-3′) and Cb-H650 (5′-GAGAAGTATGGGTGGAAAGA-3′), resulting in a 1232 bp long fragment including the entire cytochrome *b* (Cyt *b*) gene and 92 bp of flanking tRNA genes. Chromatograms were checked for quality and sequences were aligned using SeqScape 2.5 (Applied Biosystems). Sequences were deposited in Genbank (accession numbers JQ651395 to JQ652369).

Eleven microsatellite loci were successfully genotyped in 889 individuals. Ten out of the 11 primer pairs used in this work (Lsou5, Lsou8, Lsou10, Lsou34, BL1-2b, BL1-61, BL1-84, BL1-98, BL1-153 and BL1-T2) were described previously [31], [32] and one novel microsatellite locus was genotyped (BL1-36; core repeated motif: (AC)_3_; Genbank ID JQ652370) using primers BL1-36F (6FAM-5′-GATGACTGTGCGATGAATGC-3′) and BL1-36R (5′-TGTGTGTGCAGTTTGTGTGG-3′). The eleven loci were amplified through four multiplex PCRs. Amplicons were analyzed and allele sizes were scored using protocols and conditions described by [32]. All individuals with missing data (unscored loci) were discarded from the dataset and were not used for statistical analyses. To ensure reliability, ∼10% of the 889 individuals were genotyped twice, in all cases yielding identical results.

### Population genetics analyses

In order to explore the diversity of the mitochondrial gene pool, Arlequin 3.11 [33] was used to compute the number of distinct haplotypes (*k*), the gene diversity (*H*) [34], as well as three estimators of the parameter *θ* = 2*Nμ*: *θ_π_* [35], *θ_k_* [36] and *θ_S_* [37]. To explore the diversity of microsatellite data, Genepop 4.0 [38] was used to: (i) test for Hardy-Weinberg (HW) equilibrium, (ii) estimate the heterozygosity for all loci and populations, and (iii) test linkage disequilibrium (LD) among loci within populations. Levels of significance for HW and LD tests were adjusted using the false discovery rate (FDR) procedure [39]. HW deficiencies were further tested using Micro-Checker 2.2.3 [40] in order to determine the causes of departures from HW equilibrium: genuine HW disequilibrium, null alleles, scoring errors (often resulting from stuttering) or allele dropout. For all loci, the observed (*Ho*) and expected (*He*) heterozygosities were estimated for each population sample using Arlequin 3.11. For all population samples and groups of populations, the mean number of alleles per locus (*A_n_*), the average *Ho* and average *He* over loci were estimated with Arlequin 3.11. As the observed number of alleles in a sample is highly dependent on sample size, we used the rarefaction procedure [41] implemented in Adze 1.0 [42] to estimate the allelic richness (*A_r_*) [43] and the private allelic richness (*A_p_*) [41]. When several closely related populations are sampled, few alleles tend to be private to individual populations [41]. As proposed by [42], *A_p_* was therefore estimated for groups of populations within the Rhone and Danube drainages.

### Phylogenetic analyses

**Figure 2:**
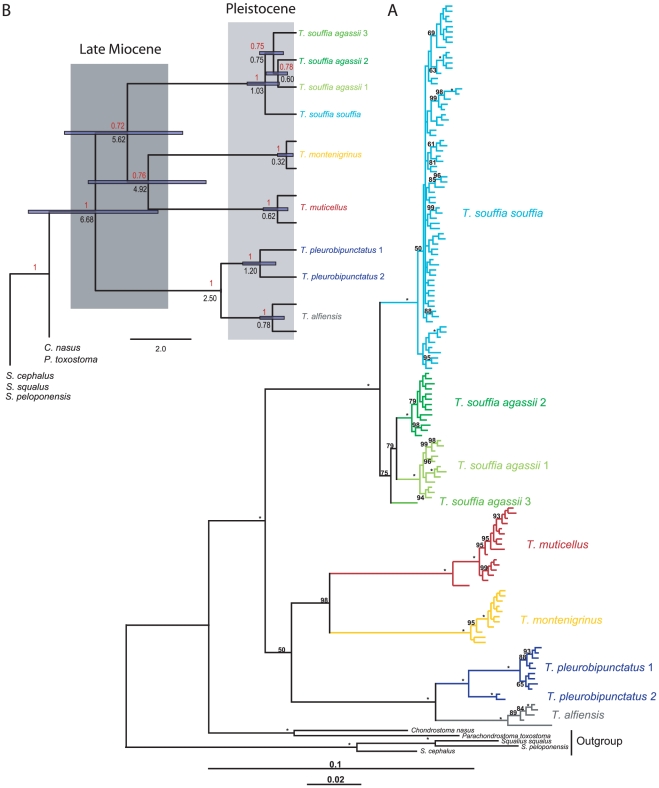
Phylogenetic analyses of mtDNA sequences. A. Phylogenetic relationships among haplotypes of *Telestes* hypothesized from Bayesian analysis using 1232 bp of mtDNA. Posterior probability*100 are indicated, with “*” corresponding to pp = 0.1 while “-” are used to indicate pp<0.5. B. Bayesian time calibrated maximum clade credibility tree using relaxed clock with the same dataset but selected terminals representing major lineages. The calibration point used was the divergence between *T. pleurobipunctatus* and *T. alfiensis*. Posterior probabilities are indicated in red on the upper left of the nodes while the modes of the posterior distributions of the age of the nodes are indicated in Myr on the lower left in black. 95% CI are indicated with blue bars centered on the nodes.

We collated 1232 bp long mtDNA sequences obtained from 970 individuals of the *Telestes* genus and from five other Leuciscinae species that were used as outgroups (Fig. 2). We selected one terminal for each haplotype and used Modeltest 3.7 [44] to obtain the substitution models that best fit each of the three codon positions of the Cyt *b* fragment and the tRNA collated flanking regions using the Akaike Information Criterion [45]. These four models (Supporting Information S1) were subsequently used for partitioned Bayesian analysis performed with MrBayes 3.1 [46] on the University of Canterbury Supercomputer (www.ucsc.canterbury.ac.nz). Bayesian analysis consisted of two independent runs of 2.0×10^7^ generations with random starting trees and 10 Markov chains (one cold) sampled every 1000 generations. Adequate burn-in was determined by examining convergence onto stationarity on bivariate plots of the split frequencies, cumulative split frequency for all splits for the two runs of the simulation and symmetric tree-difference score [47] within and between runs using Awty [48]. We considered relationships with posterior probabilities ≥0.95 to be strongly supported.

To obtain an estimate of the molecular rates of evolution in the different *Telestes* lineages, as well as a time frame for the diversification, we used a relaxed Bayesian molecular clock with uncorrelated lognormal rates (Beast 1.4.8 [49]). We calibrated the tree reconstruction using an important and well-dated geological event: the formation of the Strait of Korinthos in the late Pliocene (2.5 Myr), which separates the Peloponnesus from mainland Greece [50]. *Telestes pleurobipunctatus* from the Arachthos River and *T. alfiensis* from the Alfios River are cyprinid taxa that were separated by the formation of the Strait of Korinthos [51]. The prior distribution of the age of the node was set as a normal distribution 2.5±0.1 Myr to account for uncertainty in the gene tree divergence. Unresolved relationships among haplotypes and intraspecific lineages impact the time estimates and rates of evolution [52]. Thus, we selected one representative of each major lineage (n = 17) according to the previous Bayesian analysis. According to the estimated models that best fit the dataset (Supporting Information S1), we used a GTR+I+G model partitioned for each codon position with unlinked parameters, and six Gamma shape categories. The tree prior used the Yule Process, with a UPGMA starting tree and operators optimized for each analysis by a preliminary run of 10^6^ generations sampled every 1000 generations followed by two independent runs of the same length and sampling rate. Adequate burn-in was determined by examining a plot of the likelihood scores of the heated chain for convergence onto stationarity.

**Figure 3:**
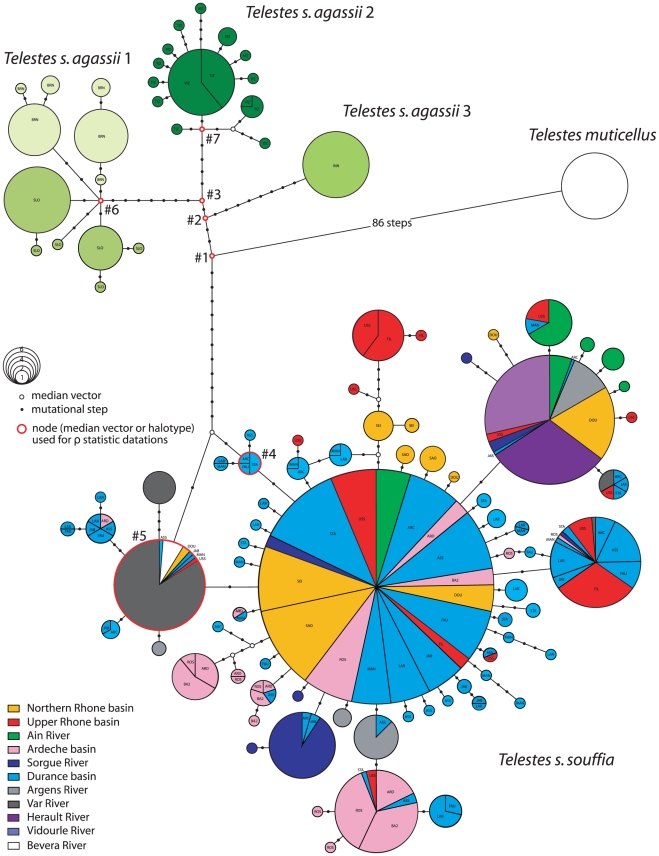
Median-Joining network of the *T. souffia* mitochondrial haplotypes. Population samples ID detailed in haplotypes charts when necessary.

Network 4.05 (Fluxus Technology Ldt, www.fluxus-engineering.com) was used to compute a Median-Joining (MJ) network [53] of haplotypes from the *T. souffia* complex (*T. muticellus* haplotype from BEV sample was used to root the network). A statistical parsimony (SP) network of haplotypes [54] was also computed using Tcs 1.21 [55]. MJ and SP network reconstructions resulted in identical topologies, except that Tcs failed to resolve the topology of some parts of the phylogeny that were resolved by the MJ network. Furthermore, several TMRCA were estimated from the Median-Joining network through Network 4.05 using the ρ statistic methodology [56], [57] and the mean calibrated evolution rate estimated as described above. We therefore only show the MJ network reconstruction (Fig. 3).

Genetic relationships between populations based on microsatellite allele frequencies were also investigated using the Cavalli-Sforza chord distance (Dc) [58] calculated with the maximum likelihood algorithm implemented in Phylip 3.69 [59]. Node support was tested using 1000 bootstrapped datasets.

### Population structure analyses

A Bayesian-based approach was used to search for the occurrence of independent genetic groups (K) in the *Telestes* microsatellite dataset (implemented in Structure 2.3.3 [60]–[63]). The burn-in length was set to 50,000 followed by 200,000 iterations within a Markov Chain Monte Carlo (MCMC). The “admixture model” was used with priors on population sampling location. Using priors on population sampling location allows genetic structure to be detected at lower levels of divergence and/or with a limited number of loci (i.e. <15 loci). Moreover, the model is not biased towards detecting non existing genetic structure [63]. The “I-model” (independent allele frequencies) was used when investigating different species, and the “C-model” (correlated allele frequencies) was used when investigating the structure of the *T. s. souffia* lineage. Ten repeats were conducted for each K value, with K = 1–15. Each individual was assigned to the inferred clusters according to the results from the simulation procedures. The way for determining the most meaningful value of K is greatly debated (see [64]). Actually, multiple biologically meaningful K values are likely for one data set depending on the biological questions asked [65]. Additionally, forcing Structure to place individuals into too few clusters often results in an inferred genetic structure that is not consistent with the evolutionary history of the populations [66]. Using CorrSieve 1.6–2 [64] we combined three approaches to determine K: (i) choosing the K value that maximizes the posterior probability of the data Ln *P*(D) [60], (ii) the Δ*K* test [67], and (iii) the Δ*Fst* test [64]. Structure does not export *Fst* values between clusters when the I-model is used. Only the first two approaches were thus used for the analyses dealing with the different *Telestes* species. Finally, *T. alfiensis* individuals were discarded from the Structure analyses since assignment tests are biased for populations of limited sample size [68].

In order to graphically summarize the allelic frequencies variation for the different populations, Factorial Correspondence Analyses (FCA), which are well suited for qualitative variables (here, genotypes) [69], were conducted using Genetix 4.05 [70].

Population differentiation was estimated by pairwise F-statistics (*Fst*) [71] calculated using Arlequin 3.11. In order to test isolation by distance (IBD) within the Rhone basin we used Mantel test [72] with 1000 permutations using Arlequin 3.11. As recommended for analyzing differentiation in elongated habitats [73], a one-dimensional model was used and we analyzed the linear relationship between *Fst*/(1−*Fst*) and (unmodified) geographical distance. A Mantel test was also conducted to explore the correlation between the microsatellite *Fst*/(1−*Fst*) and the mtDNA *Fst*/(1−*Fst*). Since null alleles were detected at two loci for a few populations (see below), FreeNA [74] was also used to calculate *Fst* including a correction for null alleles. Samples VAR, BES, HER, VID and SEI were discarded from the IBD analyses, since these are populations that are currently disconnected from the Rhone river system.

### Demographic analyses

Bayesian skyline plots (BSP) [75] permit the estimation of the effective population size (Ne) through time. We used the BSPs implemented in Beast 1.4.8 [49] for each major mitochondrial lineage in the Danube and Rhone basins. We first undertook preliminary runs for each lineage. Given previous estimations of the best-fitting model we applied a GTR+I+G model, estimated based frequencies, four Gamma categories, the previously estimated rate of molecular evolution (see before) for the species group in a strict clock model, 10 grouped coalescent intervals (m), and priors for the phylogenetic model and population sizes were uniformly distributed. These analyses were sampled every 1000^th^ iteration for 10 million generations. In a second step we undertook 40 million generation runs with bounded prior distributions and optimized tuning. Plots for each analysis were visualized using Tracer 1.4 [76].

Using mtDNA sequences, the demographic history of populations and major lineages was also investigated by computing Tajima's D (*D_T_*) [77] and Fu's *Fs* neutrality tests [78] with Arlequin 3.11. Using microsatellites, we tested for potential recent reduction in Ne through the Wilcoxon test implemented in Bottleneck 1.2 [79]: a transient excess of heterozygotes signals a recent (0.2Ne-4Ne generations) bottleneck event [80], [81]. Several alleles in our dataset testified that most loci depart from the stepwise mutation model (SMM) [82]. The infinite allele model (IAM) [83] was therefore used for Bottleneck analyses, as well as a two-phase mutation model (TPM) [84] assuming 70% of single-step mutation and variance among multiple steps of 30% (as recommended by [81]).

## Results

### Checking the microsatellites: low polymorphic loci, HWD, null alleles and LD

All the microsatellite loci were polymorphic in at least one population, and only two loci (BL1-36 and Lsou10) were found to be monomorphic in several populations. BL1-36 presented only two alleles (namely 195 and 197). Allele 195 was found in *T. alfiensis*, *T. pleurobipunctatus* and *T. muticellus* populations, while allele 197 was found in *T. montenigrinus* and *T. souffia* populations. Heterozygote individuals were only found in BEV (*T. muticellus*) and INN (*T. souffia*) populations. Lsou10 was polymorphic in *T. muticellus* (BEV, CAR and VIC), *T. montenigrinus* (POD) and *T. s. agassii* 1 (BRN and SLO) only.

After applying the FDR controlling procedure, significant departure from HW equilibrium was observed at several loci in 12 populations (including both cases of heterozygote excess and deficiency; Supporting Information S2). Significant excess of heterozygotes was observed in three populations: ARC (one locus), GLB (one locus) and INN (six loci; see below, results of Bottleneck analyses). MicroChecker analyses were conducted on populations with significant heterozygote deficiency. Neither allele dropout nor scoring errors were detected. Null alleles were detected in three loci: BL1-T2, BL1-98 and BL1-153. BL1-T2, which was originally developed from a *T. souffia* genomic library [32], presents null alleles for all three *T. muticellus* populations. BL1-98 and BL1-153 present null alleles in all eight populations with heterozygote deficiency. Null alleles are believed to have a negligible impact on assignment tests [85]. However, null alleles may bias *Fst* [74] and genetic diversity summary statistics [86]. In this study, *Fst* were estimated for *T. s. souffia* populations (IBD analyses), for which only three populations (FIL, VAR and ARD) present null alleles at one locus each. We therefore assumed a limited impact on *Fst* and genetic diversity summary statistics estimations. With regard to *Fst*, this limited impact was confirmed by the FreeNA estimation of *Fst* including a correction for null alleles: the corrected *Fst* were highly correlated to uncorrected *Fst* (Supporting Information S3).

A total of 1185 pairwise comparisons were submitted to LD analyses. Interestingly, LD was found between BL1-2b and BL1-T2 in ten populations representing four *Telestes* species or sub-species (Supporting Information S4). This pair of loci previously showed significant LD in four other European cyprinid species [87]. Since the karyology and chromosome topology are well conserved across cyprinid species (e.g. [88]), the prevalence of LD between BL1-2b and BL1-T2 may indicate a loose vicinity on the chromosome. Alternatively, BL1-2b and BL1-T2 loci may be linked to genes under selection in cyprinids. However, after FDR correction, only 19 pairs of loci exhibited LD, i.e. 1.6% of the total pairwise comparisons (Supporting Information S4), which indicates that LD is far from dominating our data set. We therefore assumed a negligible impact of LD on our analyses (see [89]).

### Population-level analyses and genetic diversity

**Figure 4:**
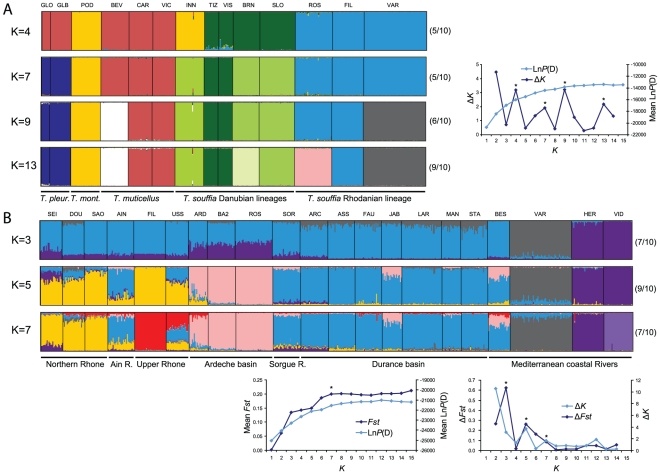
Phylogenetic and multivariate analyses of microsatellites data. Unrooted Maximum Likelihood tree based on microsatellites Dc distances (A) and FCA scatter plots for *T. s. souffia* population samples (B) and population samples representative of *Telestes* genus (C).

All but three populations (INN, HER and VID) were polymorphic for mtDNA data (Supporting Information S5). From *θ_π_* and *θ_S_* indices, BEV had an extremely high genetic diversity but harbored only two haplotypes. However, these two haplotypes were nested within two distinct species in phylogenetic reconstructions: one haplotype within *T. muticellus* (not shared with, but one mutational step from haplotypes sampled in CAR and VIC), and one haplotype within *T. s. souffia* (shared with six populations of the Rhone drainage and very frequent in the Var River) (Fig. 3). This confirms hybridization between *T. muticellus* and *T. s. souffia* in the Bevera River [90]. However, Structure failed to identify individuals with a possible nuclear genomic component from *T. s. souffia* in our BEV sample (Fig. 4A).

*θ_k_* is known to be very sensitive to Ne fluctuations, more than *H*, *θ_π_* or *θ_S_* [91]. For instance, although BEV had the highest *θ_π_* and *θ_S_*, this sample exhibited a very low *θ_k_* (0.28) when compared to most of the other *Telestes* population samples (up to 8.48 in *T. muticellus* and 12.46 in *T. souffia*). This may indicate a hybridization event associated with a sharp bottleneck event, which is also suggested by the very low *A_r_* value.

Within the Danube drainage, it is noteworthy that although mtDNA diversity and *A_r_* were similar to those encountered in the Rhone drainage (except for INN), the values of *A_p_* were about two-fold higher (even for INN). In the Rhone basin, *θ_k_* values tended to be higher in populations from the Durance basin (Southern Rhone drainage) than in most of the other more northern Rhodanian populations, whether considering individual populations or groups of populations. This pattern was similar when *A_r_* (estimated from microsatellites) was considered, except for the Ardeche basin, Sorgue River and Ain River that were very similar to the Durance basin. On the contrary, *A_p_* tended to be lower in the Durance basin (0.60) than in the northern areas of the Rhone drainage (except the Upper Rhone), with a peak of *A_p_* in the Ardeche basin (0.95). Contrasted diversity patterns were observed within the French Mediterranean coastal rivers (BES, VAR, HER and VID). Their genetic diversity indices ranged from comparable to or slightly higher than some Rhodanian populations (in BES) to very low genetic diversity (in VID).

### Phylogeography and genetic structures

A total of 140 distinct mitochondrial haplotypes were found in the entire *Telestes* dataset, with 96 in the *T. souffia* complex and 71 in *T. s. souffia* alone. Bayesian phylogenetic tree reconstruction (Fig. 2A) provided a well resolved topology with most of the posterior probabilities (pp) of the nodes between the different taxonomic entities in the *Telestes* genus being >0.95. Three major clades were recovered: (i) *T. souffia* complex comprising four highly divergent lineages, (ii) an Italian/Balkan clade with *T. muticellus*+*T. montenigrinus*, and (iii) a Greek clade with *T. pleurobipunctatus+T. alfiensis*. Nevertheless, a few ambiguities remain: the basal split among these three *Telestes* clades and the relationships among the four lineages within the *T. souffia* complex remain poorly supported. Moreover, *T. pleurobipunctatus*, *T. muticellus* and *T. alfiensis* are also each subdivided into highly divergent lineages that do not spatially overlap (except for *T. muticellus*). The same topology (except the basal split among *Telestes* clades) was recovered in the Bayesian relaxed molecular clock time calibration (Fig. 2B). Most nodes are strongly supported (pp≥0.95), except the dichotomies between the *T. souffia* complex and *T. muticellus*+*T. montenigrinus* (pp = 0.72), between *T. muticellus* and *T. montenigrinus* (pp = 0.76), between *T. s. agassii* 3 and *T. s. agassii* 1+*T. s. agassii* 2 (pp = 0.75) and between *T. s. agassii* 1 and *T. s. agassii* 2 (pp = 0.78).

**Figure 5:**
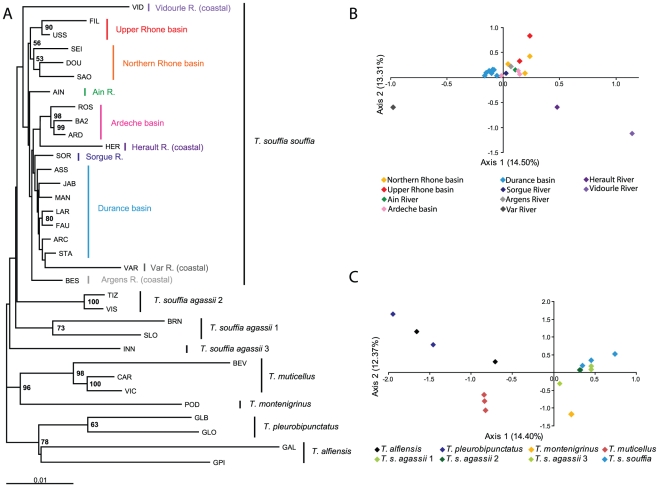
Bayesian clustering analyses of microsatellites using Structure. Estimated population structure for population samples representative of *Telestes* genus (A) and *T. s. souffia* (B). “*” indicates K values retained from Δ*K* [67] and Δ*Fst* [64] tests and when maximizing the posterior probability value [60]. Between parentheses, the number of convergent runs associated to the structure displayed.

The topology of the ML tree based on microsatellites (Fig. 5A) was congruent with the mitochondrial gene trees. The three main mitochondrial clades were also recovered: *T. muticellus*+*T. montenigrinus* (BP = 96), *T. pleurobipunctatus*+*T. alfiensis* (BP = 78) and the *Telestes souffia* complex (although BP<50) with INN (*T. s. agassii* 3) displaying basal position. Within *T. pleurobipunctatus*, the GLB and GLO populations formed differentiated clusters (BP = 63). Moreover, the 21 populations of *T. s. souffia* form a clade (although BP<50). As for the FCA conducted on populations (Fig. 5C), it clustered all three *T. muticellus* populations together on the one hand, and all but two (INN, *T. s. agassii* 3; and VAR, *T. s. souffia*) *T. souffia* populations together on the other hand.

Four values of K were found to fit the data from Δ*K* analyses of Structure runs conducted on 14 populations representative of the *Telestes* genus (Fig. 4A). However, as expected when relatively long divergence times separate the investigated evolutionary lineages [66], results are hardly interpretable with K<13: in fact, clustering is phylogenetically questionable (*T. s. agassii* 3 and *T. montenigrinus* grouped together for K = 4) and no more than half of the runs were convergent. On the contrary, for K = 13, 9/10 runs converged and all population samples were discriminated, except CAR+VIC (*T. muticellus*) and GLO+GLB (*T. pleurobipunctatus*).

Our dataset allowed us to focus further on the genetic pattern and structure of the *T. souffia* complex. From the Bayesian phylogenetic tree reconstructions (Fig. 2), the *T. souffia* complex is structured into four population segments that display non-overlapping lineages. *Telestes s. souffia* is distributed in the eastern half of France (in the Rhone basin and some Mediterranean coastal rivers). The three other clusters are distributed in distinct patches in the headwaters of the Danube River catchment with INN population harboring an exclusive lineage assigned to *T. s. agassii* 3; VIZ and TIZ in Romania assigned to *T. s. agassii* 2 and BRN and SLO from the Lim and Soca rivers assigned to *T. s. agassii* 1. From the MJ network, these Danubian clades are separated by 12 to 16 mutational steps, while they are separated from *T. s. souffia* haplotypes by 19 to 21 mutational steps (Fig. 3). The same genetic structure is observed from microsatellites using ML tree reconstruction (Fig. 5A) and using Structure (for K = 7; Fig. 4A) in the *T. souffia* complex. Furthermore, from Structure analyses, no detectable gene flow could be observed between the three Danubian headwaters (i.e. between VIS+TIZ, BRN and INN).

The most readily sampled group is *T. s. souffia* with 23 populations and 681 individuals. All these populations display some degree of haplotype sharing and the *T. s. souffia* haplotype network (Fig. 3) has a noticeable star-like shape with a major haplotype having a high frequency (∼30%, n = 192). However, most populations have private haplotypes. Moreover, some populations harbor particular features on the network and peripheral haplotypes are also strongly represented: the south-eastern population VAR display one derived haplotype (3 steps from closest haplotype) that is not found elsewhere; the northern populations USS and FIL also have two haplotypes clearly distinct from the rest of the network (4 steps from closest haplotype); and populations from the Ardeche basin share very few haplotypes with the rest of the populations. On the contrary, the south Rhone populations (Durance basin) are much diversified and their haplotypes are found all around the network.

Bayesian clustering using microsatellites revealed distinct subpopulations. Three values of K (3, 5 and 7) were retained from the analysis of the Structure runs (Fig. 4B): K = 3 and K = 5 correspond to peaks of both Δ*K* and Δ*Fst*, while K = 7 corresponds to a peak for Δ*K* and to the plateau reached by mean *Fst* and mean Ln*P*(D) values. For K = 3, the Mediterranean coastal river Var on the one hand and the rivers Herault and Vidourle on the other hand are discriminated from the rest of the Rhone basin. These three coastal rivers are characterized by very low or null mitochondrial diversity and low microsatellite diversity (see Supporting Information S5). Moreover, the FCA analysis (Fig. 5B) showed that VAR, HER and VID are extremely differentiated from the other populations. Structure therefore tended to discriminate first populations that experienced deep genetic drift during their evolutionary history. For K = 5, the two additional clusters discriminated Ardeche populations (ARD, BA2 and ROS) and populations from the North of the Rhone basin (DOU, SAO, USS and FIL) and from the South Rhodanian populations (SOR and Durance basin). Interestingly, both AIN (Northern Rhone basin) and BES (Mediterranean coastal river) are hardly differentiable from the South Rhodanian populations, and SEI (from the Seine basin) is similar to the North Rhodanian populations. For K = 7, VID was discriminated from HER and a new cluster characterized the Upper Rhone (USS and FIL). Nevertheless, the genetic structure was less clear and gene flow between the different parts of the Rhone drainage could be inferred from the results: (i) AIN sample, from the Northern part of the Rhone drainage, was hardly differentiable from the South Rhodanian populations for all K values, and (ii) some traces of gene flow from the southern and northern parts of the Rhone drainage were detectable in USS and (to a lesser extent) in ARD.

**Figure 6:**
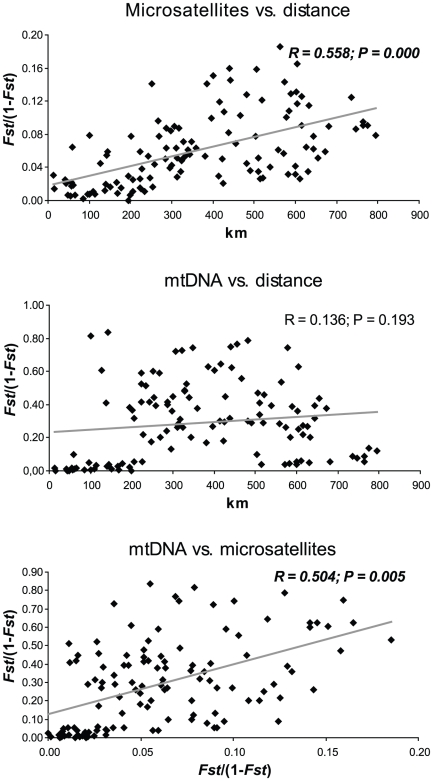
Pairwise relationships between mtDNA, microsatellites and geographical distances in the Rhone drainage. Pairwise relationship between Fst/(1−Fst) (genetic distance) vs. geographical distances (km) using microsatellites (A) and mtDNA (B) or between microsatellites vs. mtDNA genetic distances in the Rhone drainage. The correlation coefficient (R) and the P-value (P) obtained from Mantel test are reported for each bivariate analysis.

IBD was observed in the Rhone drainage using linear distances vs. *Fst*/(1−*Fst*) values estimated from microsatellites (R = 0.558; P = 0.000) (Fig. 6). Although such a correlation was not observed using *Fst* values obtained from mtDNA data (R = 0.136; P = 0.193), a Mantel test found the correlation between microsatellites *Fst*/(1−*Fst*) and mtDNA *Fst*/(1−*Fst*) to be significant (R = 0.504; P = 0.005). This minor discrepancy is probably due to the four-fold lower effective size of the mtDNA that makes this marker more prone to local genetic drift, which may have distorted the imprint of IBD. When using *Fst* corrected for null alleles (microsatellites data), Mantel tests gave similar results: R = 0.561 and P = 0.000 for linear distances vs. *Fst*/(1−*Fst*); R = 0.508 and P = 0.005 for microsatellites *Fst*/(1−*Fst*) vs. mtDNA *Fst*/(1−*Fst*).

### Molecular dates and differentiation events

The estimated calibrated rate of evolution of the *Telestes* species is 1.055E10^−2^ subs/site/Myr (95%CI 0.738–1.402E10^−2^). Our evolutionary rate is in agreement with previous estimates for Leuciscins Cyt *b* gene [51], [92], but is two-fold higher than recently estimated evolutionary rates [15], [16]. To obtain an estimation of the ages of the major cladogenic events, three different methods were used that gave remarkably similar results (Table 2).

**Table 2:** Molecular datations from mtDNA sequences.

	Coalescence time (TMRCA)							Expansion time events (BSP)	
Lineage	BEAST	95% CI	(#)^a^	ρ	95% CI	BSP	95% CI	Event 1	Event 2
*Telestes*	6.68 Myr	[4.60–8.92]		-					
(*T. muticellus*+*T. montenigrinus*) vs. *T. souffia*	5.62 Myr	[3.77–7.71]		-					
*T. muticellus vs. T. montenigrinus*	4.92 Myr	[3.00–6.92]		-					
*T. souffia* (Danube and Rhone drainages)	1.03 Myr	[0.56–1.63]	(#1)	1.01 Myr	[0.57–1.46]				
*T. souffia* (Danube drainage only)	0.75 Myr	[0.42–1.21]	(#2)	0.64 Myr	[0.37–0.91]	0.56 Myr	[0.32–0.83]		
*T. s. agassii* 1 vs. *T. s. agassii* 2	0.60 Myr	[0.28–1.00]	(#3)	0.59 Myr	[0.35–0.83]	-			
*T. s. souffia*	-		(#4)	159.2 Kyr	[3.2–315.2]	161.3 Kyr	[70.1–274.0]	∼75 Kyr	∼10 Kyr
*T. s. souffia*			(#5)	140.7 Kyr	[0.0–286.3]				
*T. s. agassii* 1	-		(#6)	125.6 Kyr	[33.5–217.7]	158.9 Kyr	[58.7–278.1]	∼60 Kyr	none
*T. s. agassii* 2	-		(#7)	110.7 Kyr	[0.0–243.6]	88.3 Kyr	[27.7–185.0]	none	∼10 Kyr

a Nodes of the MJ network used to estimate TMRCAs from ρ statistic (see Figure 3).

From time calibrated phylogenetic reconstruction, diversification in *Telestes* that corresponds with the separation between the four *Telestes* species groups (*T. souffia*, *T. muticellus*, *T. montenigrinus* and *T. pleurobipunctatus*+*T. alfiensis*) ranged from 6.7 [8.9-4.6] Myr to 4.9 [6.9-3.0] Myr ago, which corresponds to Late Miocene and matches the age of the MSC (6.0-5.3 Myr) (Table 2; Fig. 2B). Most of the divergences occurred during the Pleistocene, from 1.2 (within *T. pleurobipunctatus*) to ∼0.1 Myr ago (within *T. souffia*) (Table 2; Fig. 2B).

When focusing on the *T. souffia* complex, we obtained an estimation of the divergence between *T. s. souffia* and the other *T. souffia* lineages of ∼1 Myr ago, while the sampled Danubian lineages split between 0.7 and 0.6 Myr ago. These differentiation events fall within the Early-Middle Pleistocene transition (∼0.5–1.2 Myr), the period of some of the most severe glacial stages of the Pleistocene [93]. The TMRCA of the *T. souffia* lineages were younger with means comprised between 160 Kyr (*T. s. souffia*) and 90 Kyr (*T. s. agassii* 2), which correspond to the Late Pleistocene (Table 2).

### Demographic inferences

For demographic inferences, we focused on Rhone and Danube lineages and populations. BSPs of the *T. souffia* complex lineages revealed different demographic histories (Table 2; Supporting Information S6). In *T. s. souffia*, two phases of population expansion are likely: *Ne* seems to have increased steadily from ∼75 Kyr and a sudden burst was detected in the last 10 Kyr. On the contrary, *T. s. agassii* 2 and *T. s. agassii* 1 seem to have increased only once: while *T. s. agassii* 2 had a very stable demographic trend with a very recent increase during the last 10 Kyr, *T. s. agassii* 1 BSP indicated a population growth starting at ∼60 Kyr.

Tajima and Fu's neutrality tests suggested population expansion (both *D_T_* and *Fs* tests significantly negative) in *T. s. souffia* and *T. s. agassii* 2 populations only (Supporting Information S5). These results are in agreement with the star-like phylogeny of the MJ networks (Fig. 3) for these two lineages, which testified to demographic expansions. Interestingly, in the Rhone drainage, only the populations from the Durance basin (except FAU) showed signs of population expansion (from *D_T_* and *Fs* tests).

No signal of recent population contraction was detected from Bottleneck analyses, except in two populations: INN and BES. This indicates that anthropogenic factors have had minor impacts on the genetic diversity of the *Telestes* populations that we sampled, and that most of the observed patterns are due to the (natural) evolutionary history of the species and populations.

## Discussion

Several studies demonstrated extensive introgressions in Leuciscinae (e.g. [94], [95]) and cases of punctual hybridization involving *Telestes* species have been reported (e.g.: *Squalius peloponensis* X *Telestes alfiensis* [22], [96]; *Parachondrostoma toxostoma* X *T. s. souffia*; *Chondrostoma soetta* X *T. muticellus* [87]; *T. muticellus* X *T. s. souffia* [90]). In the present study we did detect evidence of hybridization from mtDNA but in only one river (BEV), between *T. muticellus* and *T. s. souffia*. However, we did not recover any corresponding evidence from nuclear markers (at locus BL1-36, one individual with allele 197, but no significant LD between loci or significant HW disequilibrium). This asymmetric pattern suggests a past hybridization event with the subsequent sorting of the nuclear genome from *T. s. souffia* while both mitochondrial genomes subsisted. Our sampling scheme aimed at better characterizing the introgression between *T. muticellus* and *T. s. souffia* previously detected in the Var basin [90], [97]. Although 67 individuals were analyzed, we were neither able to detect any *T. muticellus* mtDNA haplotypes in VAR, nor any evidence from microsatellites. Furthermore, any sign of hybridization between *T. s. agassii* 1 and *T. muticellus* was found in the Soca River (SLO), which represents a contact zone between these two species according to [97]. Therefore, our study strongly suggests that hybridization has only played a minor role in the evolutionary history of *Telestes*. This statement is not trivial considering the importance of this process in Cyprinid speciation [98].

### Tempo and mode of differentiation in *Telestes*

The patterns of genetic structure and diversity obtained from mtDNA and microsatellites are largely congruent. We are therefore confident that our mtDNA-based time estimates represent the *Telestes* genome evolution well.

#### The Late Miocene

The mtDNA-based estimations of divergence time between the four *Telestes* species groups (*T. souffia*, *T. muticellus*, *T. montenigrinus* and *T. pleurobipunctatus*+*T. alfiensis*) ranged from 6.7 [8.9-4.6] Myr to 4.9 [6.9-3.0] Myr. These dates are consistent with those found from 16S rDNA sequences (split between *T. souffia* and *T. muticellus*) [90], from partial Cyt *b* sequences (split between *T. souffia* and its sister species *T. beoticus* and *T. pleurobipunctatus*) [51] and from the mtDNA Control Region (divergence of *T. muticellus*, *T. souffia* and *T. turskyi*) [97]. These dates are ∼5 Myr and are very close to the end of the MSC (6.0 - 5.3 Myr). The importance of this geomorphologic event in the dispersal of the *Telestes* genus is also suggested by the distribution of the different species in independent ichthyological districts of the Mediterranean area: Rhone and Danube Rivers representing the SF and DB districts, the Po River for the PV district, the Moraca River for the AB district and the Louros and Alfios Rivers for the WG district. The Lago Mare phase that occurred at the end of the MSC may have favored the dispersal of *Telestes* across these Mediterranean districts. The Lago Mare phase then ended abruptly during the Zanclean ∼5.33 Myr [99] when the waters of the Atlantic entered the Mediterranean by the Strait of Gibraltar and re-established open marine conditions in the Mediterranean. The Zanclean therefore initiated a phase of vicariance for the *Telestes* populations that had scattered across the Mediterranean rivers.

Furthermore, during the Lago Mare, both the Mediterranean and the Parathetys were reduced to a network of lakes, among which was the Pannonian Lake (covering the current Hungary). And yet, the freshwater Parathetys drained into the freshwater Mediterranean, connecting the Adriatic/Ionian area with the forthcoming Danube drainage [5]. The split between *T. souffia* (Danubian) and *T. montenigrinus*+*T. muticellus* (both Adriatic) ∼5.6 Myr suggests that *Telestes* colonized the Danube drainage as early as the Lago Mare stage.

#### The Pleistocene

Divergences among *T. souffia* sub-species range between 0.6 and 1.0 Myr. The estimation of the divergence time between the Rhodanian *T. s. souffia* and its Danubian sister sub-species is 1.0 [0.6–1.6] Myr from time calibrated phylogeny and 1.0 [0.6–1.5] Myr from the *ρ* statistic method. These dates are consistent with the mtDNA Control Region based estimate of [97]: 1.0 [0.7–1.3] Myr. Many changes in upstream river capture involving the Danube, Rhine and Rhone drainages are recorded for the Pliocene and the Early-Middle Pleistocene transition (e.g. [100]). For example, in the Late Pliocene, waters forming the current Upper Rhine were carried out eastward and fed the Danube drainage [101], [102], at the start of the Middle Pleistocene, the waters of the current Alpine Rhine were diverted northward and fed the Rhine drainage [100]. *Telestes souffia* might have benefited a series of similar upstream river capture events to colonize the Rhone drainage from the Danube, possibly via the Rhine drainage. Moreover, the formation of periglacial lakes at the edge of glaciers are known to have transiently connected the Rhine and Rhone drainages during the LGM or the Younger Dryas promoting the dispersal of *Cottus gobio* lineages from the Rhine drainage to the Danube drainage [14], [103]. Similar periglacial lakes may also have favored the dispersal of *T. souffia* in the Rhone drainage during the Middle Pleistocene.

**Figure 7:**
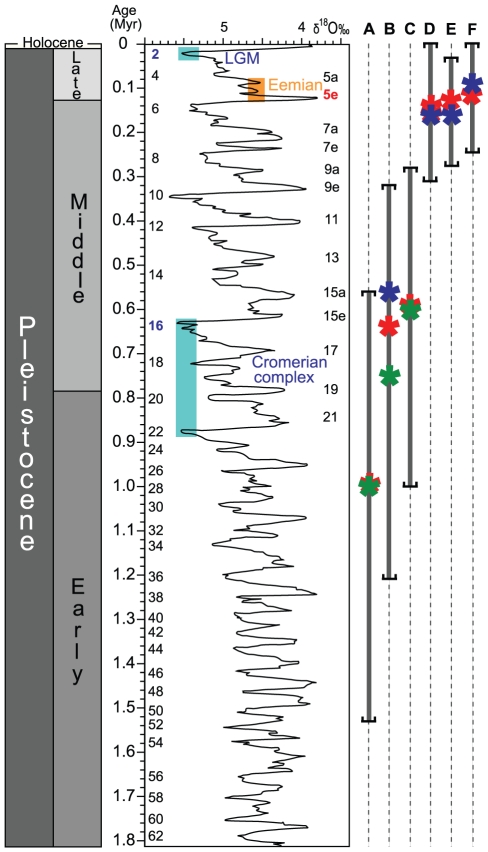
Time frame of the Quaternary period and the TMRCA obtained for *T. souffia* lineages. The mean and 95% CI maximum interval of the TMRCA obtained for *T. souffia* lineages using mtDNA sequences and MIS are indicated. A. *T. souffia* (Rhone and Danube); B. *T. souffia* (Danube only); C. *T. s. agassii* 1 vs. *T. s. agassii* 2; D. *T. s. souffia*; E. *T. s. agassii* 1; F. *T. s. agassii* 2. Green “*”, means of TMRCA obtained from BEAST; red “*”, means of TMRCA obtained from the ρ statistic methodology; blue “*”, means of TMRCA obtained from BSP methodology.

The crown ages of the different *T. souffia* lineages in the Danube are comprised between 0.7 and 0.6 Myr. *T. souffia* does not currently occur in the main course of the Danube [7]. Our data suggest this was the case even before anthropogenic modifications. In fact, the different populations in the Danube are highly differentiated and have no sign of admixture based on microsatellites or mtDNA (*T. s. agassii* 1, *T. s. agassii* 2 and *T. s. agassii* 3 are reciprocally monophyletic). The estimated TMRCAs fall within the transition between Early and Middle Pleistocene (0.5–1.2 Myr [93]), when the most extensive glaciations in the Quaternary occurred. During this period, the Northern Hemisphere experienced two of the most severe and longest glacial periods [93]: the Marine oxygen-Isotopic Stage 22 (MIS22; *c.* 870-880 Kyr) and MIS16 (*c.* 650-620 Kyr) (see Fig. 7). During glacial periods, European meandering rivers (such as the Danube) tended to adopt braided courses due to the increase of periglacial-derived gravel and sand discharge and a decrease in water flow [104]. The Early-Middle Pleistocene transition therefore may correspond to the spread of suitable habitats for *T. souffia* in the main course of the Danube. Such conditions have likely allowed the small river and cold adapted *T. souffia* to colonize downstream Danubian sub-drainages such as the Sava (including the Lim River) and Tisza river systems. Subsequent milder climate conditions, notably the interglacial MIS15 (*c.* 620-560 Kyr; see Fig. 7), have probably restricted *T. souffia* suitable habitats to the Danube peripheral tributaries, preventing further dispersals and gene flows. The glacial periods in the Middle and Late Pleistocene were less severe in amplitude and duration and, given the observed genetic structure, have unlikely permitted subsequent spread of favorable habitats and dispersal. Although times of divergences were found older (i.e. Pliocene) than in *T. souffia*, a similar pattern was also found in the cold-adapted and rheophilic *Barbatula barbatula*, for which populations were maintained in the upstream portion of the rivers [105]. As a corollary, the thermophilic *Rhodeus amarus*, a species common in lotic and lentic habitats [106], has been documented to harbor rather homogenous populations along the Danube basin. Our results therefore suggest that the Pleistocene glaciation cycles acted very differently on the dispersal and vicariance of European freshwater fish, as a function of their ecology. Accumulating detailed specific patterns may allow the inference of general evolutionary histories related to groups of species with similar ecological features.

The TMCRAs for *T. s. souffia*, *T. s. agassii* 1 and *T. s. agassii* 2 populations are all very similar (Table 2; Fig. 7), with means ranging from ∼160 Kyr to ∼90 Kyr. They straddle the Eemian interglacial period (127-117 Kyr [107]) that is increasingly considered as critical in the establishment of the genetic pattern of some extant or recently extinct species [20]. The Eemian started with a very warm stage (MIS5e, when mean surface temperatures were at least 2°C warmer than present [108]). Subsequent events of demographic expansion were detected during the cooler Eemian stages that followed MIS5e for *T. s. souffia* and *T. s. agassii* 1 (at ∼75 and ∼60 Kyr respectively; Table 2, Supporting Information S6). This suggests that, both in Rhone River and Danube River, the climatic and ecological conditions during the MIS5e have been quite unfavorable for the cold-adapted *T. souffia*, while cooler climates promoted demographic expansion.

The *T. s. agassii* 1 population from the Soca River constitutes the only known occurrence of this lineage outside the Danube drainage. Upstream river capture seems the most likely cause of this current pattern. However, the age of this event remains ambiguous. The mean TMRCA of the *T. s. agassii* 1 (involving both Danubian and Adriatic populations) is ∼120-160 Kyr old, but Danubian and Adriatic populations share a population expansion ∼60 Kyr ago. Despite lack of precision, molecular dating suggests that the upstream river capture responsible for the occurrence of *T. s. agassii* 1 in an Adriatic river likely dates back to the Late Pleistocene. Upstream river catchment has been previously suggested for *T. muticellus* [109]. As supported by nuclear data, such a geomorphologic event favored exchanges between the Po river system and some Ligurian coastal rivers. As well, it could explain the co-occurrence of a divergent mtDNA lineage in the Po River [110].

#### The Holocene and anthropogenic factors

Demographic expansion was detected in *T. s. agassii* 1 and *T. s. souffia* ∼10 Kyr (Table 2, Supporting Information S6). The LGM (MIS2) spanned 26.5-19.0 Kyr [111]. After a transient stage of global warming, climate reverted to glacial conditions during the Younger Dryas (12.8-11.5 Kyr [112]). The age for the latest expansion events within *T. souffia* lineages therefore suggest that the end of the Younger Dryas (rather than the end of the LGM) was critical (as suggested for *Cottus gobio* [103]) in promoting demographic expansion.

For *T. s. souffia* (Rhone drainage), the processes involved in this expansion could be investigated in more detail given the completeness of the sampling. When all populations are considered, a clear signal of demographic expansion is detected using mtDNA (from BSP analysis and neutrality tests). However, only samples from the Durance River (in the southern Rhone basin) exhibit signs of population expansion when considered individually. This result suggests a glacial refugium during the LGM/Younger Dryas in the South of the Rhone drainage. This southern Rhone refugium hypothesis is further supported by higher mtDNA diversity in Durance (with respect to *θ_k_*) than in the other parts of the range. Considering microsatellites, the pattern of *A_p_* also supports a range expansion from the Southern Rhone: higher *A_p_* values in populations from the Northern Durance River (Ardeche basin, Ain River, Upper Rhone and Northern Rhone) fit the so called allele surfing model [113]. This model of range expansion assumes that, during the colonisation stages, the allele frequencies within the wave of advance could be sharply modified and that new alleles have a higher probability of being retained and to propagate. For *T. s. souffia*, we can hypothesize a demographic and range expansion that would have started in the Southern Rhone drainage at the beginning of the Holocene.

Along with this major glacial refugium in the Southern Rhone basin for *T. s. souffia*, several elements suggest that two potential microrefugia also co-occurred during the LGM/Younger Dryas. As a matter of fact, the Ardeche basin and the Upper Rhone are currently connected to the rest of the Rhone basin but hold the genetic signature of past isolation: high microsatellite *A_r_* values and multiple divergent and private mtDNA haplotypes. As previously recognized for some other taxa [114], [115], microrefugia could have actually played a critical role during the post-glacial recolonisation processes.

Interestingly, the Bayesian clustering analysis (Fig. 4B) does not distinguish SOR from the Durance basin and suggests that AIN and USS constituted examples of mixed populations between northern and southern clusters. These results indicate that the Rhone seems to have acted as a corridor, probably until very recently, among *T. s. souffia* populations that are isolated today. In fact, before the Rhone River was channelized and fragmented during the 19th and the 20th centuries, *T. s. souffia* occurred in the main course of the Rhone River [116].

The differentiation pattern and lack of genetic diversity observed in the isolated populations of the French Mediterranean coastal rivers (VAR, HER, VID) could be explained by recent downstream connections during phases of sea level drops linked to the glacial periods. A subsequent rise in sea level at the beginning of the Holocene would have led to isolation of the populations and genetic differentiation. MtDNA haplotype sharing, the Bayesian clustering analysis (K = 3 and K = 5) and (to a lesser extent) the FCA, suggest that HER and VID stemmed from the same source population. However, this scenario is unlikely for the Var River population. In fact, the topology of the seafloor in this region does not support the possibility of a past connection between the Var and the Rhone rivers, even when the sea was at its lowest level. Alternatively, the topography of the Var drainage and of the Verdon drainage (Durance basin) supports possible episodic upstream connections either linked to the Alpine orogenesis or to periglacial lakes. Moreover, these three coastal river populations clearly experienced sharp genetic drift, suggesting that the connection was very limited in time and that a founder effect characterized their settlement processes.

However, these scenarios were not supported for the Argens coastal river (BES). The fact that BES is both indistinguishable from the Durance basin (based on Bayesian clustering approach) and has experienced a recent bottleneck (Fig. 4B, Supporting Information S5) supports a very recent colonisation involving quite few individuals. In fact, Argens basin was connected to Durance basin in the mid-20^th^ century by a channel devoted to irrigation and human water consumption, the *Canal de Provence* [117], [118], which could have led to the colonisation of the Argens River by *T. s. souffia*. A similar scenario involving human influence could also be invoked for the pattern observed in SEI, which is indistinguishable from the other Northern Rhone drainage populations, DOU and SAO (based on Bayesian clustering approach applied to microsatellites). In the mid-19th century, the Seine, Rhone, Rhine and Loire basins were connected through the construction of a network of navigation channels. These channels have been previously proven to have allowed some cyprinid species to cross basins. They were notably instrumental in the invasion of French hydrographic systems by *Chondrostoma nasus* [119], [120].

#### A case of parapatric differentiation?

Although they are found in the same river system, the two populations of *T. pleurobipunctatus* from the Louros River exhibit an important genetic differentiation based on microsatellites and harbor very distinct mitochondrial lineages. These results confirmed previous work that used allozymic data [22] and indicate the existence of reproductive isolation between the two lineages. In fact, these lineages are found in two distinct habitats of the Louros river system: GLO was sampled in the mainstream river whereas GLB was sampled in flood plain ponds that skirt the mainstream river. Our results therefore support a parapatric differentiation stemming from ecological specialization of the two lineages. This evolutionary process has started in the Pleistocene, likely from 1.2 Myr as suggested by the age of the two *T. pleurobipunctatus* mtDNA lineages (Fig. 2B, Table 2). Additionally, the case of *T. pleurobipunctatus* in the Louros River illustrates that, although allopatric differentiation (vicariance) seems to have been the major process, parapatric differentiation may have played a non-negligible role in the *Telestes* genus evolution.

### Conclusions

We were able to establish several clear links between major historical events and the genetic differentiation in *Telestes*. These events range from geomorphologic, climatic to anthropogenic factors and date back from Late Miocene to Holocene. Most of them constituted a series of alternations between dispersal and vicariance that critically impacted the evolution of the *Telestes* genus. Additionally, the dynamics of recolonisation of a species from glacial refugial zones in a hydrographic system are still poorly understood (but see [121]). The detailed analysis of the Rhone drainage suggested that a combination between range expansion and IBD can explain current patterns of differentiation at finer geographical scales. Our study also suggests that several climatic oscillations (MIS16/MIS15, Eemian, LGM/Younger Dryas and Holocene) have had a critical impact on the demography and the range of several *Telestes* lineages. Moreover, in the current context of global climate change, our data related to the Eemian may suggest a potential risk of population decline for *T. souffia* in the near future (the mean global temperature during MIS5e having been at least 2°C higher than the current mean temperature). These results could constitute important foundation information for improving the conservation strategies for European freshwater fishes.

Furthermore, the existence of general patterns of colonisation for the European freshwater fishes (as inferred for terrestrial fauna, e.g. [12]) were recently questioned (e.g. [13], [122]). We believe that the approach developed in this study, establishing fine relationships between time, patterns and processes, should assist in determining the existence of such general patterns.

## Supporting Information

Supporting Information S1 Models of mtDNA sequence evolution.
(XLS)

Supporting Information S2 Results from HW equilibrium tests conducted on microsatellites.
(XLS)

Supporting Information S3 Pairwise relationship between uncorrected *Fst* and *Fst* corrected for null alleles (*Fst*_c_).
(PDF)

Supporting Information S4 Results from LD tests conducted on microsatellites.
(XLS)

Supporting Information S5 Genetic diversity and demographic parameters obtained from mtDNA and microsatellites data.
(XLS)

Supporting Information S6 Bayesian Skyline Plots for *T. s. souffia*, *T. s. agassii* 1 and *T. s. agassii* 2.
(PDF)
