# Supplementary material for: From Late Miocene to Holocene: Processes of Differentiation within the Telestes Genus (Actinopterygii: Cyprinidae)
Source: PLoS One. 2012 Mar 29;7(3):e34423. doi: 10.1371/journal.pone.0034423 (PMC3315529; doi:10.1371/journal.pone.0034423)
Supplement: Supporting Information S3 — Pairwise relationship between uncorrected Fst and Fst corrected for null alleles (Fst c). (PDF) [file pone.0034423.s003.pdf]

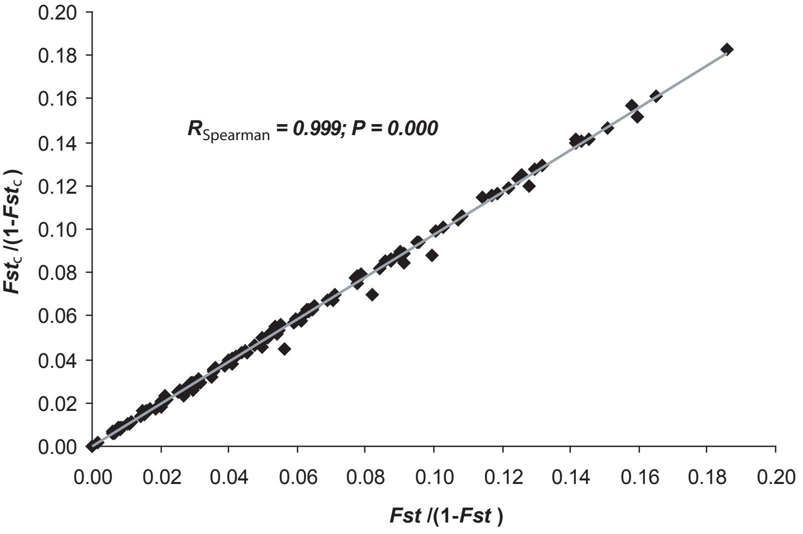

Supporting Information S3. Pairwise relationship between uncorrected *Fst* and *Fst* corrected for null alleles (Fst_c).
