# Supplementary figures and images for: From Late Miocene to Holocene: Processes of Differentiation within the Telestes Genus (Actinopterygii: Cyprinidae)
Source: PLoS One. 2012 Mar 29;7(3):e34423. doi: 10.1371/journal.pone.0034423 (PMC3315529; doi:10.1371/journal.pone.0034423)

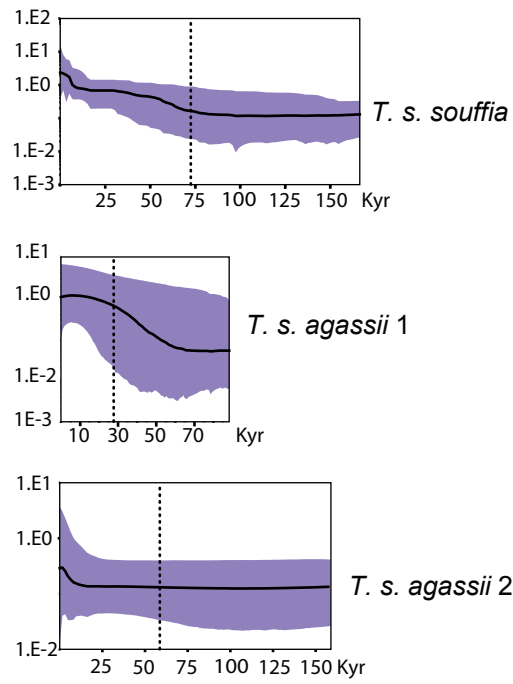

**Supporting Information S6.** Bayesian Skyline Plots for *T. s. souffia*, *T. s. agassii* 1 and *T. s. agassii* 2.

Supplement: Supporting Information S6 — Bayesian Skyline Plots for T. s. souffia, T. s. agassii 1 and T. s. agassii 2. (PDF) [file pone.0034423.s006.pdf]
